# Resection Probability Maps for Quality Assessment of Glioma Surgery without Brain Location Bias

**DOI:** 10.1371/journal.pone.0073353

**Published:** 2013-09-06

**Authors:** Philip C. De Witt Hamer, Eef J. Hendriks, Emmanuel Mandonnet, Frederik Barkhof, Aeilko H. Zwinderman, Hugues Duffau

**Affiliations:** 1 Neurosurgical Center Amsterdam, VU University Medical Center, Amsterdam, The Netherlands; 2 Department of neurosurgery, Hôpital de Lariboisière, Paris, France; 3 Department of Radiology and Image Analysis Center, VU University Medical Center, Amsterdam, The Netherlands; 4 Department of Clinical Epidemiology, Biostatistics, and Bioinformatics, Academic Medical Center, University of Amsterdam, Amsterdam, The Netherlands; 5 Department of Neurosurgery, Hôpital Gui de Chauliac, Centre Hospitalier Universitaire Montpellier, Montpellier, France; The Ohio State University Medical Center, United States of America

## Abstract

**Background:**

Intraoperative brain stimulation mapping reduces permanent postoperative deficits and extends tumor removal in resective surgery for glioma patients. Successful functional mapping is assumed to depend on the surgical team's expertise. In this study, glioma resection results are quantified and compared using a novel approach, so-called resection probability maps (RPM), exemplified by a surgical team comparison, here with long and short experience in mapping.

**Methods:**

Adult patients with glioma were included by two centers with two and fifteen years of mapping experience. Resective surgery was targeted at non-enhanced MRI extension and was limited by functional boundaries. Neurological outcome was compared. To compare resection results, we applied RPMs to quantify and compare the resection probability throughout the brain at 1 mm resolution. Considerations for spatial dependence and multiple comparisons were taken into account.

**Results:**

The senior surgical team contributed 56, and the junior team 52 patients. The patient cohorts were comparable in age, preoperative tumor volume, lateralization, and lobe localization. Neurological outcome was similar between teams. The resection probability on the RPMs was very similar, with none (0%) of 703,967 voxels in left-sided tumors being differentially resected, and 124 (0.02%) of 644,153 voxels in right-sided tumors.

**Conclusion:**

RPMs provide a quantitative volumetric method to compare resection results, which we present as standard for quality assessment of resective glioma surgery because brain location bias is avoided. Stimulation mapping is a robust surgical technique, because the neurological outcome and functional-based resection results using stimulation mapping are independent of surgical experience, supporting wider implementation.

## Introduction

A larger extent of resection (EOR) of diffusely infiltrative glioma is associated with increased survival [1], [2]. Therefore, resective surgery aims to maximize glioma removal while preserving functional integrity. Consequently, these two aims require integration in quality assessment of glioma surgery. Standards for quality of glioma resections have not been determined.

Several techniques are in use to improve glioma surgery. Some intend to preserve functional integrity, such as preoperative functional MRI and diffusion tensor imaging [3], [4]. Others intend to maximize glioma removal, such as fluorescence light microscopy [5] and intraoperative MRI [6]. Furthermore, intraoperative stimulation mapping, identifying functional brain regions, serves both aims [7].

To be useful for patient counseling and surgical decision-making, the impact of such techniques on the aims of resection should ideally be quantified. Reporting on the EOR has not been standardized. Several publications have reported the surgeon's intraoperative impression of completeness of the resection without radiological verification [8]–[12]. Others have reported the percentage of patients with ‘gross total tumor removal’ using radiological verification, but with varying definitions, such as “no radiological residual glioma tissue”, “less than 1 cm rim”, and “resection of at least 90% of the preoperative glioma volume” [13]–[15]. Still others have reported the mean EOR [2], [16]. Obviously, one important determinant of EOR is the tumor localization within the brain. The tumor localization in reports of EOR is often not mentioned [1], [15], or attributed in subgroup analysis of lobar involvement [12], [14] or eloquency [2].

As an alternative, here we present an approach to quantitate and statistically test differences in EOR between cohorts utilizing maps of likelihood of resection. Probability maps have previously been applied in surgical glioma patients to estimate the EOR [17] and to identify regions of plasticity [18].

In a recent meta-analysis, intraoperative stimulation mapping was associated with a reduction of permanent postoperative deficits and an increase in EOR [7]. Practical implementations of this technique have been detailed [19], [20]. It is generally assumed that successful application of this technique depends on experience of the surgical team. In this study, we demonstrate the use of resection probability maps (RPMs), exemplified by a comparison of the quality of resective glioma surgery in two surgical centers with differential experience in stimulation mapping of 15 and 2 years, respectively.

## Materials and Methods

### 2.1 Patients

Consecutive patient cohorts were established at two tertiary referral centers for neuro-oncological surgery, one in Montpellier, France, considered the senior surgical team, and the other in Amsterdam, The Netherlands, considered the junior team. The patients from Montpellier have been previously described [18]. The patients from Amsterdam were operated between 2009 and 2011.

Approval of the study protocol by the institutional review board (VU University Medical Center, Medical Ethical Research Committee, Amsterdam, The Netherlands) and informed consent was not required according to the Dutch health law of February 26, 1998 (amended March 1, 2006), i.e. Wet medisch-wetenschappelijk onderzoek met mensen (WMO; Medical Research Involving Human Subjects Act), Division 1, Section 1.2, because subjects are not subjected to procedures and are not required to follow rules of behavior outside routine clinical care. Furthermore, the data were analyzed anonymously.

From each center patients over 17 years of age were consecutively included (1) with diffusely infiltrative glioma of WHO grade II or infiltrative glioma that consists largely of WHO grade II characteristics with an anaplastic focus of mitotic activity, resulting in WHO grade III diagnosis according to histopathological examination, (2) in whom the MRI fluid attenuated inversion recovery (FLAIR) hyperintense signal abnormality was the target for resection, (3) who had no prior radiotherapy to avoid misinterpretation of MRI FLAIR hyperintensity, and (4) had a 3 to 6 month postoperative MRI available. Patients from the senior surgical team cohort with a (supra)total resection [21] were excluded from this analysis for two reasons. First, the current methodology for the probability maps is based on the postoperative resection cavity and residual tumor (described in detail in section 2.3), so that the security margin in supratotal resections would be considered tumor tissue as part of the resection cavity, while this tissue appeared normal on MRI. Second, the senior surgical team cohort was constituted for a previous study that focussed on the limitations of brain plasticity [18], and accordingly included resections with residues. The junior surgical team had no (supra)total resections.

This is a retrospective cohort, because postoperative imaging was analyzed after surgery with a standardized protocol that was not established before the interventions of these patients.

Postoperative deficits were determined by neurological examination, and considered permanent at three months postoperative.

### 2.2 Surgical technique and differences between surgical teams

Surgical teams consisted of a neurosurgeon, a neuroanesthesiologist, and a neuropsychologist or speech therapist. The two teams followed near identical surgical procedures. Preoperative functional MRI and diffusion tensor imaging fiber tracking was available. Anesthesiological conditions consisted of the awake-asleep-awake procedure [19]. After discontinuation of sedation cortical stimulation mapping was applied with a bipolar stimulator using 1.5–4.0 mA of biphasic 60 Hz block pulse (Nimbus; Newmedic, Labege, France) during execution of applicable paradigms for language, motor, sensory, visuospatial and visual field performance. Glioma infiltrated tissue was resected, preserving the identified functional regions. Resection was alternated with subcortical stimulation to identify functional white matter pathways at the resection cavity margins. The surgical microscope and intraoperative ultrasound were utilized as required. Intraoperative MRI was not available.

The main difference between the patient cohorts was the years of experience in stimulation mapping of the surgical team. HD completed his neurosurgical residency in 1995, and started using this technique in 1996 adding up to 15 years of experience for this patient cohort after approximately 400 glioma resections using stimulation mapping. PW completed his neurosurgical residency in 2008, and started working with this technique in 2009 after three months of education in functional mapping by HD, adding up to 2 years of experience for this patient cohort, describing the first patients here. An additional minor difference between the surgical teams consisted of glioma localization before resection using ultrasound by the senior team, and using neuronavigation by the junior team. Another minor difference was that the senior team used the DO80 and the junior team used the Snodgrass picture set for intraoperative language assessment. Furthermore, the referral pattern of patients for complex glioma resections to the senior surgical team combined with the exclusion of (supra)total resections from their cohort likely contributed to more complex glioma locations in the senior surgical team cohort.

### 2.3 Resection probability map processing

**Figure 1:**
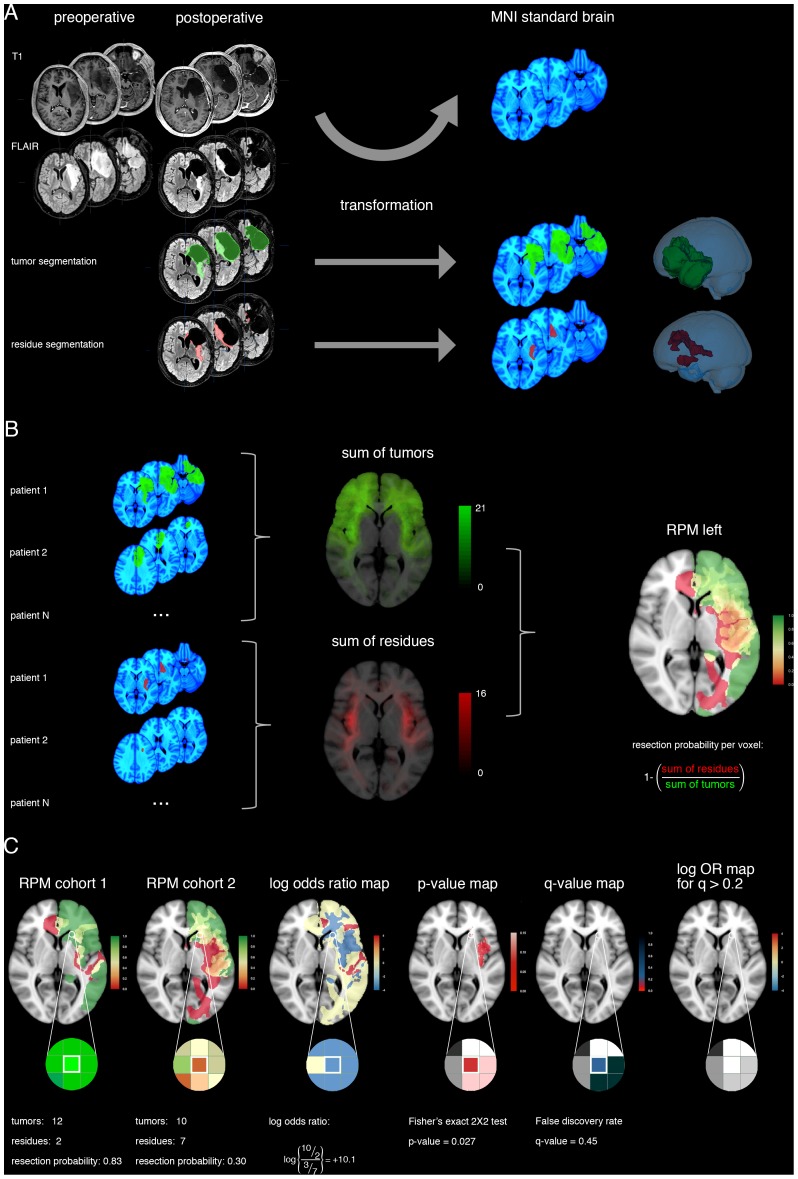
RPM processing and statistical comparison. (A) For a patient, the glioma tumor volume (green) and residual volume (red) are obtained by segmentation of postoperative MRI FLAIR sequence images. Then the transformation from the patient's brain to standard brain space (MNI152 shown in blue) is calculated by non-linear registration, and the segmented volumes are transformed accordingly. A 3D reconstruction of the brain and volumes in standard brain space are shown. (B) For each patient cohort, summation of the tumor volumes at each voxel provides a tumor localization map (green). Similarly, summation of the residual volumes at each voxel provides a residual localization map (red). The number of patient volumes contained in a voxel is provided as green-scale and red-scale legends. The probability of resection at each voxel is calculated by dividing the number of patients without residual tumor by the number of patients with a glioma at a specific voxel. This results in a resection probability map (RPM) for each hemisphere (here shown for left-sided tumors) to discern contralateral from ipsilateral tumor extension. The RPM legend represents the probability of resection from 0 (red) via 0.5 (yellow) to 1 (green). (C) For statistical comparison of RPMs between cohorts, here the left-hemisphere results are plotted as an example for a single voxel. From these RPMs, the log odds ratio map, adjusted p-value map and q-value map are derived, as detailed in the Methods section. Legends for log odds ratios, p- and q-values are provided. As a calculation example the information for one voxel is included with a probability of resection of 10 out of 12 patients, and 3 out of 10 patients, respectively. This results in a log odds ratio of 10.1, an adjusted p-value of 0.027, and a q-value of 0.45, which is above the arbitrary threshold of 0.2 for false discovery and therefore considered an indifferential resection result at the outlined voxel.

The methodology for the RPMs is outlined in Fig. 1. For each patient, a 3 to 6 month postoperative MRI FLAIR and 3D heavily T1-weighted gradient-echo pulse sequence were available from various 1.5T scanners, including GE Signa, Siemens Avanto, Sonata and Symphony, and Philips Intera. All MR images were acquired with 1 mm isotropic resolution.

First, the MR FLAIR dicom images were imported in iPlan 3.0 software (BrainLAB AG, Feldkirchen, Germany). The residual glioma and the resection cavity were segmented using the smartbrush function with interpolation. Postoperative FLAIR hyperintense signal regions were compared with postoperative diffusion-weighted images and preoperative FLAIR-weighted images for identification of residual glioma. The compound image of residue and cavity was considered the tumor volume. Segmented volumes were verified and adjusted in reconstruction planes by two observers (EH and PW). The binarized segmented volumes were exported for further analysis.

Second, FLAIR- and T1-weighted dicom images were converted to NIfTI format and linearly registered in Slicer 3.6 software [22] using the BRAINSfit module with Rigid and Affine algorithm. Then, the T1 volume was non-linearly registered to the Montreal Neurological Institute brain template, i.e. standard brain space [23]. For the non-linear registration, the BRAINSfit pipeline of Rigid, ScaleSkewVersor3D, Affine and BSpline algorithms was applied with tumor masking. The registration result was visually verified for all patients and repeated after grid size trimming, when necessary.

Third, the segmented volumes were converted to NIfTI format, and warped according to the patient-specific transformation from FLAIR to T1 and from T1 to standard brain space, i.e. the 152 T1 normal brain template of 1 mm available from the Montreal Neurological Institute (MNI). This resulted in residue and tumor volumes of all patients that were aligned to standard brain space with 1×1×1 mm voxels for further analysis.

Fourth, at each of the 1.8 million voxels, that cover the standard brain space, the probability of resection was calculated for each voxel by dividing the number of patients without a residue by the summary of patients having a glioma at that voxel. There were 1.3 million informative voxels. To discern contralateral from ipsilateral residues, RPMs were compiled for left- and right-sided tumors. The left-sided RPM covered 703,967 informative voxels, and the right-sided RPM 644,153. This resulted in four high-resolution RPMs for likelihood of resection within the brain for each hemisphere and each cohort.

### 2.4 Statistical analysis of resection probability maps

The proportion of resected glioma tissue was compared voxel-wise between cohorts using a two-sided Fisher exact test [24], because of the presence of small numbers in our observations. Two characteristics concern the statistical analysis of this type of neuroimaging data: spatial dependency of voxels measurements and multiple testing.

The spatial dependency of the data was addressed by estimating the empirical null-distribution of Fisher exact p-values for each voxel based on a permutation with relabeling of patients to one of two cohorts [25]. As the full permutation is beyond computational limits, a subset of 2,000 randomizations was randomly drawn without replacement to provide a reasonable estimate [25]–[27]. The adjusted p-value per voxel was calculated by relating the observed p-value to the empirical null-distribution of 2,000 randomized p-values under the null-hypothesis of exchangeability of cohorts [26], [28], [29].

Multiple hypothesis testing is inherent to voxel-wise comparison of neuroimaging data and was controlled here using the false discovery rate, as is commonly applied in functional MRI analysis [30]–[32]. The false discovery rate was expressed as q-value for each of 1.3 million informative voxels. These q-values are interpreted as the proportion of voxels with false differential probability of resection among all voxels declared differentially resected, when the adjusted p-value of a particular voxel is called significant. To obtain the q-value for a voxel the estimated number of false discoveries was divided by the number of voxels declared significant. The estimated number of false discoveries given a specific adjusted p-value threshold was determined from the empirical null-distribution of 2,000 randomizations per voxel [33]. The number of voxels declared significant was determined from the distribution of observed adjusted p-values. Given the explorative nature of the research question, a liberal false discovery threshold of 0.2 was chosen, [34] i.e. 20% of voxels, which are declared differentially resected, are in fact similarly resected [35]. This resulted in adjusted p- and q-value maps of differentially resected brain regions that were superimposed on the standard brain template for anatomical interpretation.

Statistical procedures were custom written in R (v2.15.1; R: A Language and Environment for Statistical Computing, R Core Team, R Foundation for Statistical Computing, Vienna, Austria, 2012) using fisher.test()from the base package, functions from the oro.nifti package (v0.3.5) for import, export and display [36], and functions from the data.table package (v1.8.4; http://datatable.r-forge.r-project.org) to store and retrieve calculations for large data sets efficiently. Because of RAM and disk space requirements for randomization calculations, commercial cloud computation running R on Ubuntu was used for final analysis (Amazon Web Services, Elastic Cloud Computing).

### 2.5 Other statistical analysis

Distributions of age and glioma volume were compared between cohorts using the Mann Whitney U test. Gender, lateralization, localization, neurological outcome, extent of resection over 90%, and residual volume less than 10 mL were compared using the Fisher exact test. The extent of resection was calculated as percentage of preoperative glioma volume that was resected. The extents of resection and residual glioma volumes were compared between cohorts using Wilcoxon signed-rank test. Statistical procedures were executed in R software.

## Results

The cohort treated by the senior team consisted of 56 patients, and that of the junior team of 52 patients. Distributions of age, gender, preoperative glioma volume, lateralization and lobular localization were comparable (Table 1).

**Table 1:** Patient characteristics.

	senior cohort	junior cohort	p value
number of patients	56	52	
mean age (range)	39 (18–62)	41 (18–73)	0.692
female, n (%)	30 (54%)	20 (38%)	0.127
mean glioma volume in ml (range)	62 (5–181)	63 (5–174)	0.971
left hemisphere, n (%)	27 (48%)	23 (44%)	0.703
lobular localization, n (%)			0.663
frontal	27 (48%)	24 (46%)	
temporal	15 (27%)	11 (21%)	
parietal	7 (12%)	11 (21%)	
insular	7 (12%)	6 (12%)	

### 3.1 Neurological outcome

None (0%) of 56 patients in the senior team's cohort had permanent neurological deficits, one (1.9%) of 52 patients in the junior team's cohort had a permanent hemiparesis grade 4. A 66-year-old man with a 132 mL oligodendroglioma with anaplastic foci, considered WHO grade III, in the right insula had resection-induced ischemia of the internal capsule. There was no indication of different neurological outcome between cohorts (p = 0.482).

### 3.2 Resection outcome

A standard for comparison of resection outcome between cohorts is unavailable. Usually resection results of cohorts are reported as extent of resection or postoperative residual volume. To contrast our new method using RPMs, we tested differences using this customary reporting. We used arbitrary thresholds of 90% extent of resection and 10 mL postoperative residual volume as previously reported [2].

The median extent of resection was 66% in the senior team's cohort, and 92% in the junior team's cohort (p<0.001). Seventeen (14%) of 56 patients in the senior team's cohort had an extent of resection over 90%, and 30 (58%) of 52 patients in the junior team's cohort (p<0.001).

The median postoperative residual volume was 12 mL in the senior team's cohort, and five mL in the junior team's cohort (p<0.001). Twenty-three (41%) of 56 patients in the senior team's cohort had a postoperative residual volume of less than 10 mL, and 38 (73%) of 52 patients in the junior team's cohort (p<0.001).

**Figure 2:**
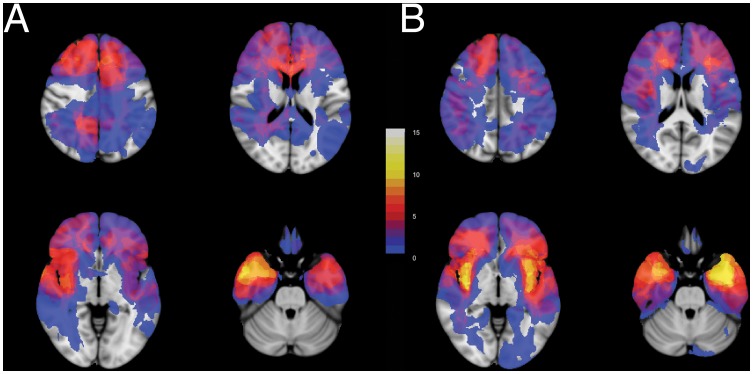
Glioma locations within the brain are dissimilar between cohorts. Four transversal sections from (A) the junior team's cohort, n = 52, and (B) the senior team's cohort, n = 56, are shown superimposed on standard brain space (MNI152). More gliomas are located in the left insula and left temporal lobe in the senior team's cohort. More gliomas are located in the left supplementary motor cortex and right temporal lobe in the junior team's cohort. The legend refers to the number of patients with glioma tissue at a voxel. See Movie S1 for all transversal sections.

Based on conventional reporting of resection results, the resection outcome of the junior team seemed to be favorable compared to the senior team. Nevertheless, this apparent difference is subject to bias from location within the brain. Even though no sign of differential lobular locations was noted (Table 1), glioma locations were dissimilar between cohorts (Fig. 2 and Movie S1). The senior team's cohort involved considerably more complex locations, such as the left insula and left temporal lobe, compared with the junior team's cohort, which involved less complex locations more often, such as the left supplementary motor cortex and the right temporal lobe. These differences in brain locations motivated our new method for comparison of resection results using RPMs.

### 3.3 Resection probability maps comparison

**Figure 3:**
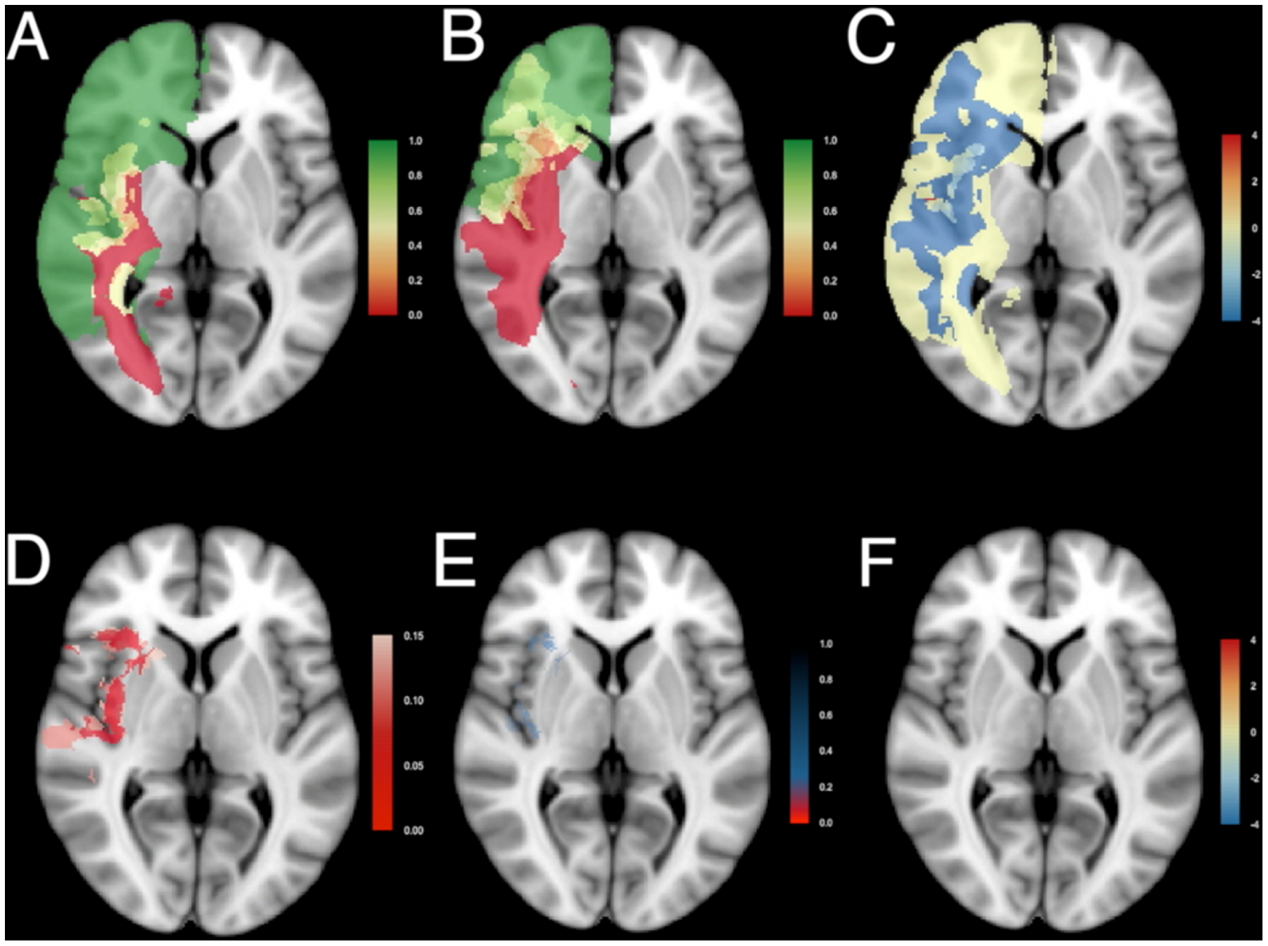
Resection probability maps for right-sided gliomas. Results comparing (A) the junior surgical team, n = 29, and (B) the senior surgical team, n = 29, are shown superimposed on standard brain space (MNI152). A probability of 0 (red) represents locations where tumor was never resected, and a probability of 1 (green) represents locations where tumor was resected in all patients. An intermediate probability (yellow) represents locations where glioma was removed in a subset of patients. (C) Relative differences in probability of resection as log odds ratio. (D) The adjusted p-value map adjusted by the empirical null-distribution to address spatial dependency of voxels. Values less than 0.15 are plotted in shades of red. (E) The q-value map to address multiple testing. Values below 0.2 are plotted in shades of red, values between 0.2 and 0.8 in shades of blue. (F) Differences in probability of resection as log odds ratio for voxels with a q-value less than 0.2 demonstrate similar resection results between the two patient cohorts. Results are superimposed on a transversal section at z = 0 of MNI152. See Movie S2 for all transversal sections.

**Figure 4:**
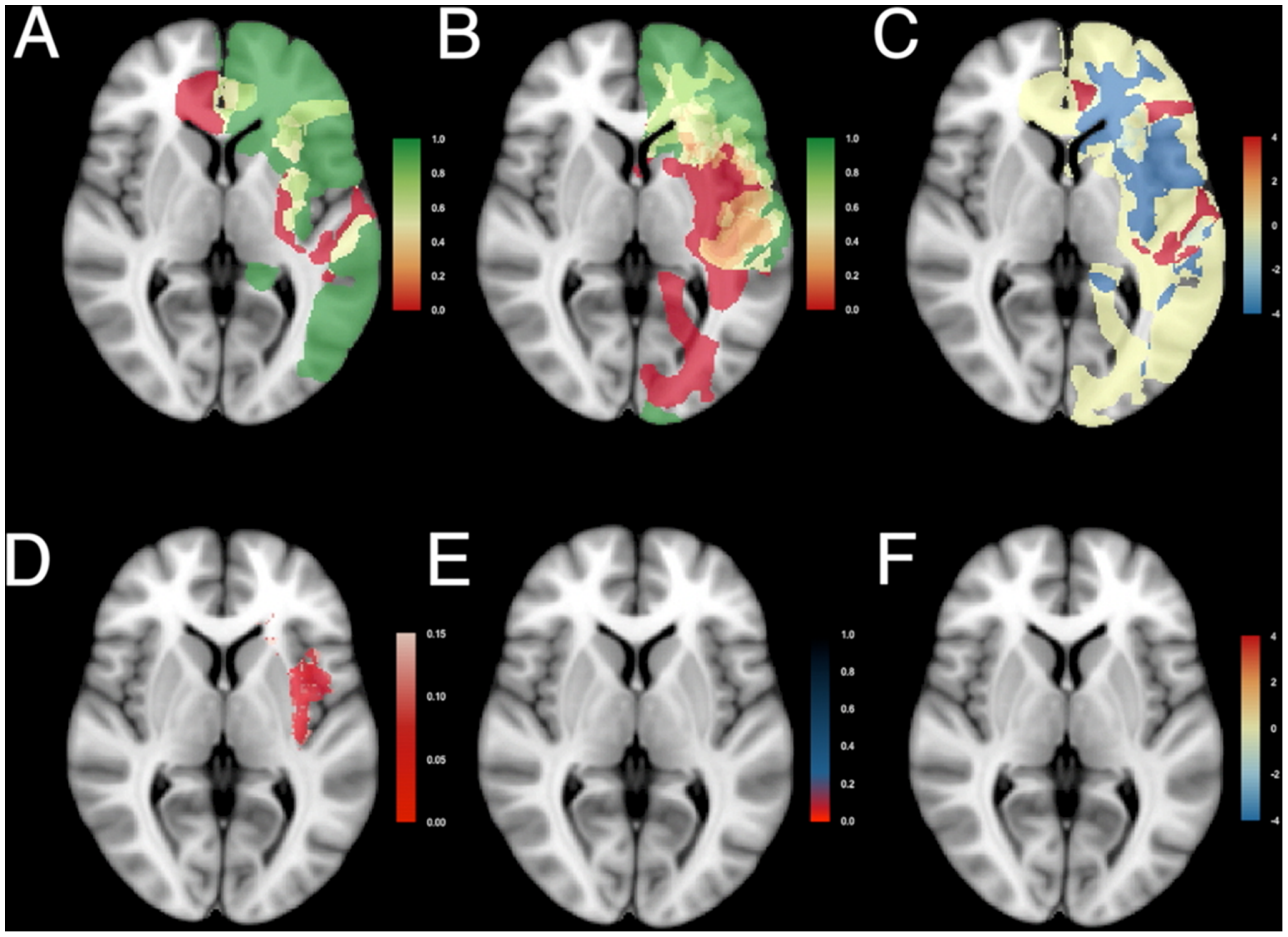
Resection probability maps for left-sided gliomas. Results comparing the junior surgical team, n = 23, and the senior surgical team, n = 27. Coding and legends as in Fig. 3. See Movie S3 for all transversal sections.

The RPMs for right-sided (Fig. 3 and Movie S2) and left-sided (Fig. 4 and Movie S3) gliomas of the junior team (Fig. 3A and 4A) and the senior team (Fig. 3B and 4B) are shown. For the right, 29 patients were included from the junior team, and 29 from the senior team; for the left, 23 from the junior team and 27 from the senior team. The RPMs are very similar. Regions that were generally resected (green) included the prefrontal cortex, the right lateral frontal cortex, large regions of the right temporal lobe, the left anterior temporal lobe, and the anterior part of the corpus callosum. Regions that could generally not be removed (red) included the anterior perforating substance involving the lenticulostriatal arteries, the corticospinal tract, the optic radiation, the left arcuate fasciculus, and the left posterior insula. Regions that were occasionally resected, depending on the stimulation mapping, included the left lateral frontal cortex, the insula and the superior parietal lobule.

The odds ratio between resection probabilities was calculated at each voxel as relative resection difference. To obtain a symmetrical distribution the logarithm of the odds ratio was considered (Fig. 3C and Fig. 4C), providing negative values for probabilities of resection favoring the junior team (blue), and positive values favoring the senior team (red). Log odds ratios were in general close to zero. The probability of resection of some regions seemed higher for the senior team, such as the right supplementary motor area, the superior part of the right posterior insula, the right superior parietal lobule, and the lateral part of the left postcentral gyrus. Whereas in other areas the probability of resection seemed higher for the junior team, such as the right temporal lobe, the right inferior insula, the right dorsolateral premotor cortex , and the left anterior insula.

These observed relative differences were then voxel-wise tested for significance, enabling an adjusted p-value map. This takes account of the spatial dependence, but not of the correction for multiple testing. According to Fig. 3D and Fig. 4D, several regions are candidates for differential probability of resection (red), such as the uncus, the insular cortex, and the right supplementary motor area.

Subsequently, the false discovery rates were calculated and presented as q-value maps taking multiple statistical testing into account (Fig. 3E and Fig. 4E). This demonstrated, after correction for multiple statistical testing, in red none (0%) of 703,967 voxels in left-sided tumors being differentially resected, and 124 (0.02%) of 644,153 voxels in right-sided tumors. These were located in the right insular cortex and temporal stem. In Fig. 3F and Fig. 4F the log odds ratios are displayed for the voxels with q-values less than 0.2. The RPMs demonstrated similar probability of resection between the cohorts.

## Discussion

The main findings of this study are that (1) RPMs provide a quantitative volumetric method to compare EOR between cohorts, and (2) stimulation mapping is a robust surgical technique, because both the neurological outcome and functional-based resection results using stimulation mapping are independent of surgical experience.

### 4.1 Explanations for residual glioma after resective surgery

Residual glioma tissue is frequently observed after resective surgery for infiltrative gliomas. Postoperative residues can have several explanations: intentional and unintentional causes need to be considered. First, intentional residues involve regions where glioma cannot be removed because critical functional structures have been infiltrated, such as the corticospinal tract or arcuate fascicle. Extending the resection to these regions would result in permanent deficits. Second, intentional residues may involve regions where critical vasculature is surrounded by glioma, such as the lenticulostriatal arteries at the medial margin of insular gliomas. Extending the resection beyond these arteries could result in deprived vascularization and ischemia of critical functional regions, such as the internal capsule, resulting in permanent deficits. Third, intentional residues may involve regions nearby critical functional structures. A safety margin of glioma is then accepted, anticipating on avoidance of transient deficits, as postresection edema, contusion and reversible hypoperfusion at the margin of the resection cavity will not involve critical regions. Fourth, unintentional residues can occur because glioma was not recognized during surgery. Attempts to distinguish infiltrative glioma from normal brain during surgery rely on microscopical appearance, tissue consistency, (functional) anatomical context, image-guided or ultrasound navigation, and intraoperative MRI. Each of these techniques is subject to false negative observations. Fifth, unintentional residues can occur because of early cessation of surgery, resulting in a multistage procedure. This includes prolonged postictal loss of function after epileptic seizures or patient fatigue during stimulation mapping, loss of surgical orientation, unexpected longevity of the procedure, or anesthesiological circumstances.

### 4.2 Similar RPMs using stimulation mapping

Our approach for comparison of surgical performance between centers using RPMs is empirical, because no distinction could be made between intentional and unintentional residues. The observed similarity of resection results by surgical teams with short and long experience clearly supports the robustness of stimulation mapping in resective glioma surgery. This also demonstrates that stimulation mapping serves reproducibility of glioma removal, which is of benefit for patients when applied by a well-trained surgical team even with short experience in advanced mapping techniques. This stands as a testimony to the integrity of neurosurgical education.

### 4.3 RPMs as a new perspective on surgical eloquence

The RPMs may not provide definitive information to discern those regions that can universally be removed safely and those that always require preservation. These maps enable group analysis of resection results, rather than guide surgical resection for an individual patient. Widespread regions with intermediate probability of resection substantiate this argument. In addition to unintentional residues in our results, probably introducing some imprecision, both false positive and false negative information likely exist. First, green regions may not necessarily remain green. Resected regions may have resulted in cognitive deficits, which were not detected by standard neurological and neuropsychological examination. Second, red regions may not necessarily remain red. Regions with glioma infiltration, that were preserved due to positive findings during stimulation mapping, may not have resulted in permanent deficits, should these been resected. The stimulation paradigm can be subject to false positive findings due to electrical current spread at distance from the stimulator [37]. Alternatively, positive findings could have detected brain regions that would have been functionally compensated.

**Figure 5:**
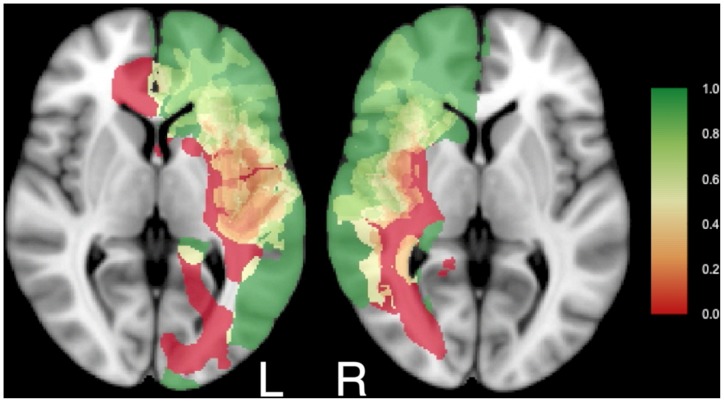
The minimal common brain for the left and right hemisphere. Results of resection probability from the junior (n = 52) and senior team (n = 56) were combined. Legend as in Fig. 3A and 3B. See Movie S4 for all transversal sections.

RPMs of residual glioma detected by stimulation mapping do hold information on functional brain organization. Some brain regions were never resected in any patient. These regions have been collectively referred to as the ‘minimal common brain’ [18], which is represented in red in Figs. 3 and 4. The similarity of the RPMs between our two cohorts adds support to this being a genuine biological concept, rather than an artifact. Therefore a new and more detailed perspective on the ‘minimal common brain’ is provided based on 108 patients in Fig. 5 and Movie S4. The ‘minimal common brain’ can be considered a universal trunk of the brain that is essential for human brain functioning. Then, some other brain regions exist that can be included in the resection only in a subset of patients, represented in yellow in Figs. 3–5 and Movie S4. This is likely due to diversity, compensation or relocation of brain function, which can be considered as plasticity of the brain. The combination of the minimal common brain and regions that can be subject to plasticity are customary referred to as ‘eloquent’, i.e. critical functional, regions. A generally accepted definition of eloquence for resective brain surgery is however lacking. Several proposals for ‘surgical eloquence’ have been published, [38]–[40] but these are based on surface anatomy with presumed functionality, and not on functional outcome. This may not be the most reliable source for surgical planning [41]. Furthermore, localization information is often limited to lobe involvement or categorization by eloquence in reports of surgical outcome [5], [6], [42]. Our results consolidate localization in high resolution avoiding brain location bias or presumptions on ‘eloquence’. This clearly demonstrates that surgical eloquence is rather a dynamic continuum that requires individualized assessment by intraoperative stimulation mapping, than a static on/off phenomenon that can be applied preoperatively to all patients with a glioma. We are confident that this approach facilitates discussions on surgical strategy to pinpoint regions with differential probability of resection.

### 4.4 RPMs for quality assessment of glioma surgery

In addition, these RPMs could serve as an instrument for quality assessment of glioma surgery without bias from brain location. Increasing attention is drawn for objective criteria to evaluate surgical outcome. In various surgical fields the high-complex, low-volume care seems to benefit from subspecialist care [43]–[48]. Outcome criteria are lacking for glioma surgery. The customary reporting of EOR or residual glioma volume is of no value for comparison of resection results because these are subject to considerable bias from brain location. RPMs can be useful to compare surgical cohorts without this bias and to achieve standards for neurosurgical care.

### 4.5 Consideration

The strength of our approach is in the methodology based on generally-accepted imaging analysis techniques, such as robust image registration, adjusted p-value calculation based on an empirical null-distribution, and the false discovery rate. A weakness of our approach is that inclusion criteria were not prospectively applied, and surgical protocols differed slightly. Furthermore, to detect smaller differences in probability of resection, larger patient cohorts are required in general. The dependency of regional voxels and multiple testing hampers a formal sample size calculation, but as an example the minimal detectable difference in probability of resection at a voxel is approximately 30% with 30 patients per cohort given a power of 0.8 and a significance level of 0.10.

### 4.6 Practical implications

Our findings can have several implications for clinical practice. First, brain stimulation mapping as an advanced neurosurgical technique is demonstrated to be effective and safe in resective glioma surgery after short education. As such, this supports a wider implementation of this useful technique by surgical teams after appropriate training. Second, RPMs can provide a detailed perspective on brain eloquence in glioma patients. Third, the RPMs can provide quantitative measures for quality assessment of resective glioma surgery without bias from brain location.

## Supporting Information

Movie S1 Glioma locations within the brain presented in transversal sections for (A) the junior surgical team and (B) the senior surgical team.
(MOV)

Movie S2 **Resection probability maps for right-sided gliomas comparing results from the junior surgical team and the senior surgical team for all transversal sections.** Coding and legends as in Fig. 3.
(MOV)

Movie S3 **Resection probability maps for left-sided gliomas comparing results from the junior surgical team and the senior surgical team for all transversal sections.** Coding and legends as in Fig. 3.
(MOV)

Movie S4 Minimal common brain in red and brain regions subject to plasticity in a subset of patients in yellow based on 108 patients for the left (L) and right (R) hemisphere. The legend refers to the probability of resection between 0 (red) and 1 (green).
(MOV)
